# Salinity-tolerant larvae of mosquito vectors in the tropical coast of Jaffna, Sri Lanka and the effect of salinity on the toxicity of *Bacillus thuringiensis* to *Aedes aegypti* larvae

**DOI:** 10.1186/1756-3305-5-269

**Published:** 2012-11-22

**Authors:** Pavilupillai J Jude, Tharmatha Tharmasegaram, Gobika Sivasubramaniyam, Meena Senthilnanthanan, Selvam Kannathasan, Selvarajah Raveendran, Ranjan Ramasamy, Sinnathamby N Surendran

**Affiliations:** 1 Department of Zoology, Faculty of Science, University of Jaffna, Jaffna, 40000, Sri Lanka; 2 Department of Chemistry, Faculty of Science, University of Jaffna, Jaffna, Sri Lanka; 3 Department of Pathology, Faculty of Medicine, University of Jaffna, Jaffna, Sri Lanka; 4 Department of Geography, Faculty of Arts, University of Jaffna, Jaffna, Sri Lanka; 5 Institute of Health Sciences, Universiti Brunei Darussalam, Gadong, BE, 1410, Brunei Darussalam

**Keywords:** *Aedes aegypti*, *Bacillus thuringiensis*, Dengue, Jaffna, Mosquito vectors, Salinity, Sri Lanka

## Abstract

**Background:**

Dengue, chikungunya, malaria, filariasis and Japanese encephalitis are common mosquito-borne diseases endemic to Sri Lanka. *Aedes aegypti* and *Aedes albopictus*, the major vectors of dengue, were recently shown to undergo pre-imaginal development in brackish water bodies in the island. A limited survey of selected coastal localities of the Jaffna district in northern Sri Lanka was carried out to identify mosquito species undergoing pre-imaginal development in brackish and saline waters. The effect of salinity on the toxicity of *Bacillus thuringiensis israelensis* larvicide to *Ae*. *aegypti* larvae at salinity levels naturally tolerated by *Ae*. *aegypti* was examined.

**Methods:**

Larvae collected at the selected sites along the Jaffna coast were identified and salinity of habitat water determined in the laboratory. The LC_50_ and LC_90_ of *B*. *thuringiensis* toxin, the active ingredient of a commercial formulation of the larvicide BACTIVEC®, were determined with *Ae*. *aegypti* larvae. Bioassays were also carried out at salinities varying from 0 to18 ppt to determine the toxicity of *Bacillus thuringiensis* to fresh and brackish water-derived larvae of *Ae*. *aegypti*.

**Results:**

Larvae of four *Anopheles*, two *Aedes*, one *Culex* and one *Lutzia* species were collected from brackish and saline sites with salinity in the range 2 to 68 ppt. The LC_50_ and LC_90_ of *B*. *thuringiensis* toxin for the second instar larvae of *Ae*. *aegypti* in fresh water were 0.006 ppm and 0.013 ppm respectively, with corresponding values for brackish water populations of 0.008 and 0.012 ppm respectively. One hundred percent survival of second instar fresh water and brackish water-derived *Ae*. *aegypti* larvae was recorded at salinity up to 10 and 12 ppt and 100% mortality at 16 and 18 ppt, yielding an LC _50_ for salinity of 13.9 ppt and 15.4 ppt at 24 h post-treatment respectively for the two populations. Statistical analysis showed significantly reduced toxicity of *B*. *thuringiensis* to fresh and brackish water-derived *Ae*. *aegypti* larvae at high salinities.

**Conclusion:**

A variety of mosquito vectors of human diseases undergo pre-imaginal development in brackish or saline waters in coastal areas of the Jaffna district in northern Sri Lanka. Salinity has a small but significant negative impact on the toxicity of *B*. *thuringiensis* toxin to *Ae*. *aegypti* larvae at salinity levels where *Ae*. *aegypti* larvae are found in the environment. This has implications for the use of *B*. *thuringiensis* toxin as a larvicide in brackish waters.

## Background

*Aedes aegypti* (Linnaeus) and *Ae*. *albopictus* Skuse are the established vectors of dengue and chikungunya in populated areas worldwide [1-3]. Dengue is of major public health concern in many tropical and semi-tropical countries [1]. In the tropical Jaffna district of northern Sri Lanka, there were 400 cases of dengue with 4 deaths in 2011 [4] and an epidemic of chikungunya in the period 2006 to 2007 [5]. Malaria has been historically endemic in Sri Lanka but its incidence has drastically declined in recent years [6]. Other important mosquito-borne diseases prevalent in Sri Lanka are filariasis [7] and Japanese encephalitis (JE) [8].

Larval source reduction is the primary means of controlling dengue in Sri Lanka and worldwide [1]. A commercial liquid formulation (BACTIVEC ®) that contains spores and toxin crystals of *Bacillus thuringiensis israelensis* H-14 is used in Sri Lanka by the Ministry of Health as a larvicide for the control of dengue vectors. Although various commercial formulations of *B*. *thuringiensis* toxin are widely used in many countries for control of different mosquito vectors [9], it is used only for *Aedes* control in Sri Lanka.

Recent studies in some coastal areas of Jaffna and the eastern Batticaloa districts of Sri Lanka show that both *Ae*. *aegypti* and *Ae*. *albopictus* naturally undergo pre-imaginal development in brackish water collections in the environment [10] including frequently-used domestic wells [11]. Water with <0.5 ppt or parts per thousand, 0.5–30 ppt and >30 ppt salt are termed fresh, brackish, and saline respectively as in previous studies [10]. Such brackish water habitats may constitute unappreciated sources of dengue vectors in tropical coasts worldwide [12]. The over-exploitation of ground water from aquifers for agricultural, domestic and industrial use, has led to increasing salinisation of groundwater in the Jaffna peninsula [13]. In this context, a limited larval survey was performed in selected coastal localities in the Jaffna district to record mosquito species undergoing pre-imaginal development in brackish and saline waters. The impact of salinity levels that *Ae*. *aegypti* larvae tolerate in their habitats on the toxicity of a commercial formulation of *B*. *thuringiensis israelensis* H-14 toxin (BACTIVEC^R^) to *Ae*. *aegypti* larvae was also examined.

## Methods

### Mosquito collections

**Figure 1:**
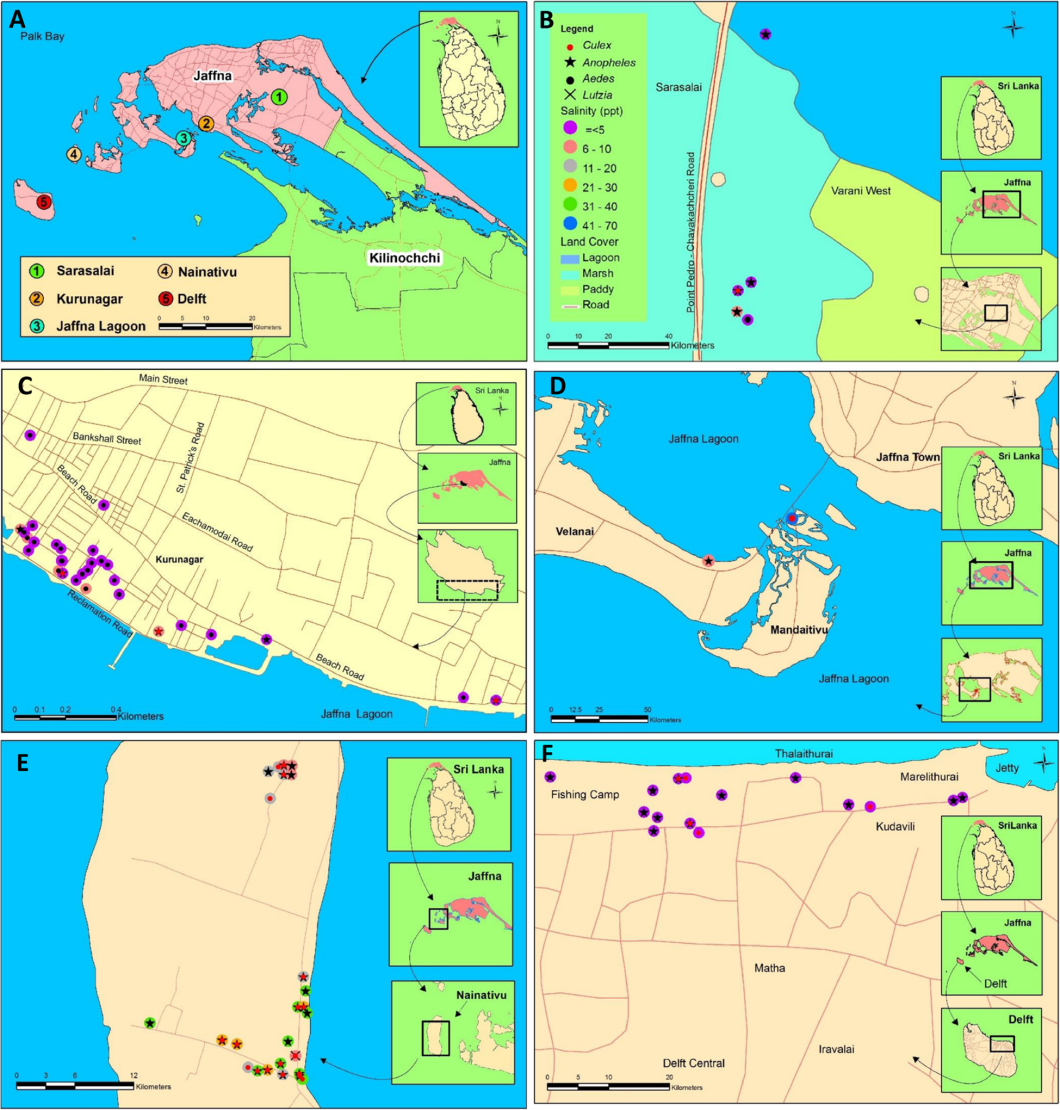
**Map showing larval collection sites in the Jaffna district of Sri Lanka.A**. Jaffna district. **B**. Sarasalai mangrove marsh. **C**. Kurunagar coast. **D**. Pannai bridge mangrove marsh. **E**. Nainativu island. **F**. Delft island.

Mosquito larvae were collected at five coastal locations *viz*. Sarasalai, Kurunagar, Pannai bridge, Delft island and Nainativu island (Figure 1A-F) in the Jaffna district during the period from August 2011 to May 2012. Kurunagar was selected based on the previous reports [10,11] that pre-imaginal stages of dengue vectors are found in brackish water in the area. Two populated islands off the Jaffna coast *viz*. Delft and Nainativu were chosen randomly for studying islands. Additionally, two readily accessible mangrove locations *viz*. at Sarasalai and Pannai bridge were selected from four other mangrove sites in the district. Collections were carried out monthly at each site. Three hundred and fifty ml capacity dippers were used to collect larvae from disused boats, pits, domestic wells, ponds and stagnant water bodies that were considered to be potentially brackish (except during the monsoon season) along the coast. Five dips per collection site on average were performed from 0800 to 1000 h. The collected larvae in water were brought to the Zoology Laboratory of the University of Jaffna at Thirunelvely, and the salinity of the water determined with a refractor-salinometer (Atago, Japan). Larvae were reared to adulthood in the respective collected water as described previously [10]. Initially for rearing purposes, *Aedes*, *Culex* and *Lutiza* larvae and later emergent adults of all mosquito species were identified using standard keys [14-16].

### Determination of LC_50_ and LC_90_ of BACTIVEC® against *Ae*. *aegypti*

From a commercial formulation of BACTIVEC® (obtained from Medical Officer of Health, Nallur), test solutions containing 0.005, 0.006, 0.013, 0.018, 0.025, 0.031, 0.063 and 0.126 ppm of *B*. *thuringiensisis* H-14 toxin (the ppm of active ingredient from the specified concentration of 0.6% in the commercial formulation) were prepared by serial dilution in a final volume of 100 ml tap water. Ten second instar larvae of *Ae*. *aegypti* from a laboratory colony that was derived from eggs deposited in fresh water ovitraps were placed in 100 ml test solution in 150 ml capacity plastic cups. Three replicates were run in parallel at each tested concentration. One hundred ml of the same tap water without BACTIVEC® was used as a control. Mortality of the larvae was determined 24 h after treatment.

A similar experiment was conducted with *Ae*. *aegypti* larvae collected from a brackish water site in Kurunagar with 10 ppt salinity and then used to establish a short-term laboratory colony with pre-imaginal development allowed to occur in tap water containing 10 ppt salt. Second instar larvae from the second generation of the colony were used in the experiment. Water of 10 ppt salinity was used in place of tap water for the experiment on brackish water-derived larvae.

### Evaluation of the toxicity of *B*. *thuringiensis* at different salinity levels

Solutions with salinity of 0, 2, 4, 6, 8, 10, 12, 14 and 16 ppt solutions were prepared by adding tap water to sea water as described previously [10]. Second instar larvae of *Ae*. *aegypti* from a laboratory colony derived from eggs oviposited in fresh water were used for this experiment. Ten larvae were introduced into each concentration of test solution in 100 ml tap water in 150 ml capacity plastic cups. Tap water alone was used as control. In one set of tests, 0.018 ppm of *B*. *thuringiensis* toxin (the lowest tested concentration that was found to cause 100% mortality among second instar larvae of fresh and brackish water-derived *Ae*. *aegypti*) was introduced into all test solutions of varying salinity. Test solutions without BACTIVEC® were used in a second set of tests. Three replicates were run in parallel at each salinity level for tests with and without BACTIVEC®. Mean larval mortality was determined at 24, 48 and 72 h post-treatment. Larvae were fed twice a day with powdered fish meal pellet for the duration of the experiments. A similar experiment at salinities of 0, 2, 4, 6, 8, 10, 12, 16 and 18 ppt was conducted with brackish water-derived *Ae*. *aegypti* larvae. Mortality in second instar larvae was determined at 24, 48 and 72 h post-treatment with and without BACTIVEC®.

### Statistical analysis

The required LC_50_ and LC_90_ values with 95% confidence intervals were determined by Probit analysis. The toxicity of *B*. *thuringiensis* at different salinity levels for each time interval was determined by a two-way ANOVA. The statistical significance of differential survival of fresh water and brackish water-derived *Ae*. *aegypti* larvae in the presence of BACTIVEC® at the salinity levels of 10, 12, 14 and 16 ppt was determined using the Student’s t-test. All analyses were done using Minitab statistical software (Minitab Inc, PA, USA).

## Results

**Figure 2:**
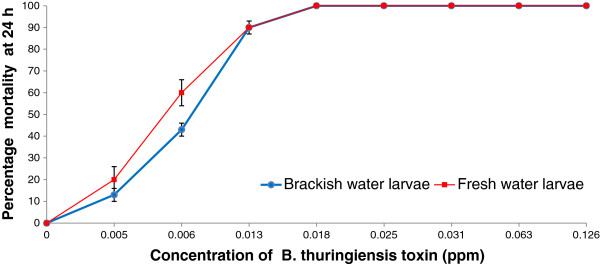
**Toxicity of *Bacillus thuringiensis israelensis *H-14 toxin against fresh and brackish water-derived second instar *Ae. aegypti *larvae at 24 h post-treatment.** The bars show standard errors of the mean.

Larvae of four *Anopheles*, two *Aedes*, one *Culex* and one *Lutzia* species were collected from brackish and saline sites with salinity in the range of 2 to 68 ppt. Details of the collected larvae and their habitats are presented in Table 1. The LC_50_ and LC_90_ of *B*. *thuringiensis* for the second instar larvae of fresh water-derived *Ae*. *aegypti* were 0.006 (95% confidence interval or CI: 0.003 - 0.009) ppm and 0.013 (95% CI: 0.009 – 0.021) ppm respectively. For 10 ppt brackish water-derived *Ae*. *aegypti*, the LC_50_ and LC_90_ values were 0.008 (95% CI: 0.006- 0.011) ppm and 0.012 (95% CI: 0.010 – 0.019) ppm respectively (Figure 2). These results do not demonstrate a significant difference in sensitivity to BACTIVEC® in the two *Ae*. *aegypti* populations.

**Table 1:** Brackish water breeding mosquitoes in Jaffna district

**Species**	**Transmitted diseases**/**medical significance**	**Location of larval collection**	**Period of larval collection**	**Nature of habitat** (**numbers of sites with larvae**)	**Average larval number per site in 350ml**/ **dip**	**Salinity range of brackish water habitats with larvae** (**ppt**)
*Aedes aegypti*	Dengue, Chikungunya	Kurunagar	2011 Sep – 2012 May	Used well (27)	6	2-9
		Kurunagar	2011 Sep	Barrel (1)	35	10
*Aedes albopictus*	Dengue, Chikungunya	Sarasalai	2012 Jan	Tube near brackish water mangrove marsh (1)	10	4
		Sarasalai	2012 Jan	Battery box in brackish water mangrove marsh (1)	8	4
*Anopheles subpictus*	Malaria	Delft	2012 Feb-April	Pond (2)	14	2-6
		Nainativu	2012 April	Used well (20)	17	4-39
		Passaiyoor	2011 Aug	Disused boats (2)	4	15-18
		Kurunagar	2011 Sep	Used well (3)	7	2-6
		Sarasalai	2012 Jan	Pit in brackish water mangrove marsh (2)	11	10-12
		Pannai bridge	2012 May	Brackish water mangrove marsh (1)	3	10
*Anopheles barbirostris*	Malaria	Delft	2012 April	Used well (3)	4	5-6
		Sarasalai	2012 Jan	Pit near brackish water mangrove marsh (1)	2	4
		Nainativu	2012 April	Used well (1)	3	15
*Anopheles varuna*	Malaria	Delft	2012 Jan-April	Used well (11)	4	2-4
*Anopheles culicifacies*	Malaria	Delft	2012 Jan-April	Used well (3)	3	2-4
		Sarasalai	2012 Jan	Brackish water mangrove marsh (1)	2	4
*Culex sitiens*	Japanese encephalitis, Ross River fever and filariasis	Kurunagar	2011 Sep-	Disused boats (1)	35	20
		Kurunagar	2011 Sep-2012 Jan	Used wells (2)	28	2-6
		Nainativu	2011 Aug - 2012 May	Used wells (18)	24	2-39
		Sarasalai	2012 Jan	Pit near brackish water mangrove marsh (1)	6	12
		Delft	2012 March	Pond (5)	9	4-6
		Pannai bridge	2012 May	Brackish water mangrove marsh (1)	5	68
*Lutzia fuscanus*	Not a vector but feeds on larvae of mosquito vectors	Nainativu	2011 Oct	Used well (1)	5	10

**Figure 3:**
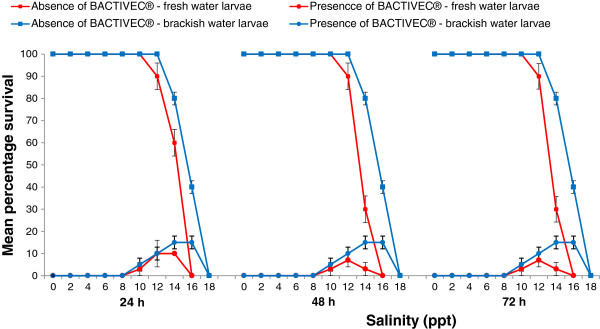
**Influence of salinity on the toxicity of 0.018 ppm *Bacillus thuringiensis israelensis *H-14 toxin on fresh and brackish water-derived second instar larvae of *Ae. aegypti *at 24 h, 48 h and 72 h post-treatment.** The bars show standard errors of the mean.

The effects of *B*. *thuringiensis* toxin at varying levels of salinity on the survival of fresh and brackish water-derived *Ae*. *aegypti* second instar larvae are presented in Figure 3. With increasing salinity in the absence of *B*. *thuringiensis* toxin, 100% survival of second instar fresh water-derived *Ae*. *aegypti* larvae was recorded at salinity up to 10 ppt and 100% mortality at 16 ppt with LC _50_ and LC_90_ values of 13.9 (95% CI: 13.1-14.8) and 15.4 (95% CI: 14.5 – 17.8 ppt respectively at 24 h post-treatment (Figure 3). Statistical analysis revealed a significant effect of salinity on the lethality of *B*. *thuringiensis* toxin at each time point (F= 49.03, P=0.00 for 24 h; F= 38.55, P= 0.00 for 48 h and 72 h). However, the brackish water-derived larvae showed 100% survival up to 12 ppt salinity and 100% mortality at 18 ppt salinity with LC_50_ and LC_90_ values of 15.4 (95% CI: 14.5 – 16.2) and 17.1 (95% CI: 16.2-19.4) ppt respectively at 24 h post-treatment (Figure 3). Although brackish water-derived *Ae*. *aegypti* larvae tended to have higher LC_50_ and LC_90_ values for salinity than fresh water-derived *Ae*. *aegypti* larvae, the differences are not significant at the P=0.5 level. Statistical analysis however, demonstrated a significant effect of salinity on the toxicity of *B*. *thuringiensis* in 10 ppt salinity at each time point (F=45.38, P=0.00 for 24 h; F=38.68, P=0.00 for 48 h and 72 h) with brackish water-derived larvae as in the case of fresh water-derived larvae. In comparison to freshwater–derived *Ae*. *aegypti* larvae, the brackish water-derived larvae showed significantly enhanced survival at 14 and 16 but not 10 and 12 ppt salinity in the presence of BACTIVEC® at 24, 48 and 72 h post-treatment (p<0.05 by the Student’s t test, Figure 3).

## Discussion

The results show that several different mosquito vectors in coastal areas of northern Sri Lanka can undergo pre-imaginal development in collections of brackish and saline water in the environment. Larvae of the known malaria vectors, *Anopheles varuna* and *Anopheles barbirostris* were detected for the first time in brackish water in Sri Lanka. Although *An*. *culicifacies s*.*l*., the major malaria vector in Sri Lanka, is recognized as a fresh water mosquito, it has been recently reported to undergo pre-imaginal development in brackish water of up to 4 ppt in eastern Sri Lanka [17]. The present findings show that this is also the case in the northern Jaffna district in Sri Lanka. *Culex sitiens* is a well-known salinity-tolerant mosquito vector of Japanese encephalitis (JE) virus [18,19] but its role in disease transmission in Sri Lanka has not established. Japanese encephalitis in Sri Lanka is considered to be mainly transmitted by *Culex tritaeniorhynchus* and *Culex gelidus* and occurrence of the disease is associated with rice cultivation and piggeries with a high incidence reported from the inland North-central province of the country [20,21]. Since pigs are also reared in coastal areas of Jaffna city, there is a potential for *Cx*. *sitiens* to transmit Japanese encephalitis in the Jaffna district.

Although morphologically characterized *An*. *subpictus s*.*l*. was detected to undergo pre-imaginal development in brackish and saline water in this study, its taxonomic status in Sri Lanka is doubtful as molecular characterization of ribosomal DNA revealed that most, if not all morphologically characterized *An*. *subpictus* species B in coastal eastern Sri Lanka are in fact *An*. *sundaicus s*.*l*. [22]. Furthermore, results of phylogenetic analysis based on the DNA sequence of the internal transcribed spacer-2 (ITS-2) of the ribosomal RNA gene of two *An*. *subpictus s*.*l*. samples collected from Nainativu island during the study period showed genetic similarity to *An*. *sundaicus s*.*l*. (sequence data not shown). *Anopheles subpictus* species B/*An*. *sundaicus* is predominant in coastal and inland areas of Jaffna peninsula [23] with a higher sporozoite rate than *An*. *culicifacies s*.*l*. [24]. Therefore, considering the well-known salinity tolerance and vector potential of *An*. *sundaicus s*.*l*. elsewhere in Asia [19], its probable presence in coastal and inland areas of the Jaffna peninsula indicates a potential for malaria transmission. Brackish water development of *Ae*. *aegypti* and *Ae*. *albopictus* has recently been demonstrated in the Jaffna and Batticaloa districts of Sri Lanka [10]. *Ae*. *aegypti* and *Ae*. *albopictus* are well known vectors of dengue and chikungunya. *Aedes albopictus* can also transmit the JE virus [19]. The present findings confirm that the two arboviral vectors are able to undergo pre-imaginal development in brackish water collections in the environment. An interesting observation is that the predatory mosquito *Lutzia fuscanus* which readily feed on other mosquito larvae, especially *Aedes* larvae (Jude, P.J., Paramsothy, S, Thavaranjith, A.C., Ramasamy, R., Surendran, S.N.; unpublished data), develops in the same brackish water as its prey in Nainativu island. This is the first report of *L*. *fuscanus* larvae in brackish water in Sri Lanka. *Lutzia fuscanus* is generally regarded to undergo pre-imaginal development in ground-water habitats with a high organic content [25]. The results suggest the *L*. *fuscanus* may naturally limit the pre-imaginal development of mosquito vectors in brackish water habitats in the Jaffna district.

A previous study that investigated the salinity tolerance of *Ae*. *aegypti* and *Ae*. *albopictus* in Jaffna district showed that the LC_50_ values for first and third instar fresh water-derived larvae of *Ae*. *aegypti* developing into adults were 11.9 and 15.5 ppt salinity respectively [10], consistent with the LC_50_ of 13.9 ppt salinity observed with fresh water-derived second instar larvae at 24 h in the present study. The corresponding values for fresh water-derived *Ae*. *albopictus* were reported to be 13.0 and 16.0 ppt in an earlier study [10]. The range of salinities observed in the habitats where *Ae*. *aegypti* and *Ae*. *albopictus* were collected in the present study are consistent with previous [10] and present laboratory results for salinity tolerance of their larvae .

The present study shows that the salinity levels that *Ae*. *aegypti* and *Ae*. *albopictus* larvae are able to tolerate in the environment has a small but significant impact on reducing the toxicity of *B*. *thuringiensis* toxin, which is commonly used for larval source reduction in Sri Lanka. Furthermore, the differential survival and LC_50_ and LC_90_ values of fresh and brackish water-derived larvae tend to suggest that *Ae*. *aegypti* may be able to adapt to salinity in its environment. The results indicate that *Ae*. *aegypti* derived from brackish water habitats may be better able to withstand the toxicity of BACTIVEC® in more brackish waters. A previous study on the effectiveness of two commercial formulations of *B*. *thuringiensis* toxin for the control of salinity-tolerant malaria vector *Anopheles aquasalis* at different salt concentrations revealed an increase in the LC_50_ for *B*. *thuringiensis* at higher salt concentrations [26]. Our results suggest that crystal formulation of *B*. *thuringiensis* toxin sprayed in the more brackish water pre-imaginal development sites of *Ae*. *aegypti* as a source reduction strategy in coastal zones may be less effective than in fresh water habitats. The nature of the interaction between salinity and the tested commercial formulation of *B*. *thuringiensis* toxin (BACTIVEC®) is not known. This could be a consequence of the effect of salinity on the toxin formulation itself or to an effect on mosquito physiology that modulates the response to the toxin. Further investigations are needed to clarify the two possibilities.

Many countries have used *B*. *thuringiensis* for malaria and dengue vector control with varying degrees of success [27-30]. However, the effect of salinity on the activity of *B*. *thuringiensis* toxin against larvae of other mosquito vector species e.g. *Ae*. *albopictus* and *An*. *sundaicus s*.*l*. in brackish and saline water also merits detailed investigation. It is possible that other commercial formulations of *B*. *thuringiensis* toxin may behave differently if the effect is due to salt on the toxin formulation itself and not larval physiology.

## Conclusion

The present study identifies several mosquito vectors of human disease that undergo pre-imaginal development in brackish or saline waters in coastal areas of the Jaffna district in northern Sri Lanka. Salt has a small but significant negative impact on the toxicity of *B*. *thurigiensis* to *Ae*. *aegypti* larvae at salinity levels where larvae are found in the environment, and this has to be taken into consideration for its use as a larvicide.

## Competing interests

The authors declare that they have no competing interests.

## Authors’ contribution

SNS, RR and MS conceived the study. PJJ and SK performed field studies, TT, PJJ and GS performed laboratory studies. SNS, TT, and MS did analysis. SR created the images. SNS and RR wrote the manuscript. All authors read and approved the manuscript.
